# Prevalence and Characteristics of Accessory Mandibular Canals: A Cone-Beam Computed Tomography Study in a European Adult Population

**DOI:** 10.3390/diagnostics12081885

**Published:** 2022-08-04

**Authors:** Giuseppe Varvara, Beatrice Feragalli, Ilser Turkyilmaz, Aurelio D’Alonzo, Fabiola Rinaldi, Serena Bianchi, Maurizio Piattelli, Guido Macchiarelli, Sara Bernardi

**Affiliations:** 1 Department of Innovative Technologies in Medicine & Dentistry, Dental School, ‘G. D’Annunzio’ University of Chieti-Pescara, 66100 Chieti, Italy; 2 Department of Medical, Oral and Biotechnological Sciences, Dental School, ‘G. D’Annunzio’ University of Chieti-Pescara, 66100 Chieti, Italy; 3 Department of Prosthodontics, New York University College of Dentistry, New York, NY 10010, USA; 4 Department of Life, Health and Environmental Sciences, University of L’Aquila, 67100 L’Aquila, Italy; 5 Center of Microscopy, University of L’Aquila, 67100 L’Aquila, Italy

**Keywords:** mandibular canal variant, bifid canals, accessory mandibular foramina, inferior alveolar nerve

## Abstract

The purpose of this observational study is to evaluate the prevalence and main characteristics of bifid canals within a European adult population, analyzing cone-beam-computed tomography (CBCT). The population study examined 300 subjects. The CBCTs were performed between 2012 and 2019, using PaX-Zenith3D with a standard protocol of acquisition. The parameters analyzed were the presence and lengths of the bifid mandibular canals. The sample included 49% male and 51% female participants. The mean age of the patients was 47.07 ± 17.7 years. Anatomical variants of the mandibular canal were identified in 28.8% of the sides and 50.3% of the patients. In 7.3% of the subjects, the anatomical variants were present bilaterally. The most frequently encountered bifid canal was Type 3 (40.5%), followed by the Type 1 canal (39.3%), the Type 2 canal (14.5%), and the Type 4 canal (5.9%), 40% on the right side and 60% on the left side. The average length of the bifid canals located on the right side of the mandible was 11.96 ± 5.57 mm, compared to 11.38 ± 4.89 mm for those measured on the left side. The bifid mandibular canal is a common anatomical variation of the mandibular canal. It is fundamental to performing an accurate preoperative evaluation using CBCT analysis to avoid and/or reduce intraoperative and postoperative complications.

## 1. Introduction

The peculiar anatomy of the oral cavity facilitates minor oral surgery procedures, since the structures to be preserved are the maxillary sinus (with its vascular supply) [1] and the alveolar nerve, well protected by the mandibular canal and highly detectable on computed tomography scans [2]. Unfortunately, some variations, less acknowledged by operators, may go undetected during surgical planning. Consequently, these add intraoperative or postoperative complications, which may be difficult to manage, such as the vascular supply of the anterior floor of the mandible [3].

The mandibular canal, generally described as single, hosting the inferior alveolar bundle, starts from the mandibular foramina, curves downward and forward, and curves back into a horizontal course below the molar roots. However, archaeological and anatomical studies have revealed the presence of variants such as bifid and trifid canals [4,5,6,7]. Chavez et al. observed that three distinct lower dental nerves innervate three distinct groups of lower teeth: incisors, deciduous molars, and permanent molars; these nerves tend to fuse during the embryonic developmental period to form a single bundle, and the incomplete fusion can lead to the formation of bifid and trifid canals [8].

The content of the bifid and trifid canals has been the subject of study and discussion over the years, confirming the content of the neurovascular bundle from the posteromedial portion of the mandibular ramus [4].

**Figure 1:**
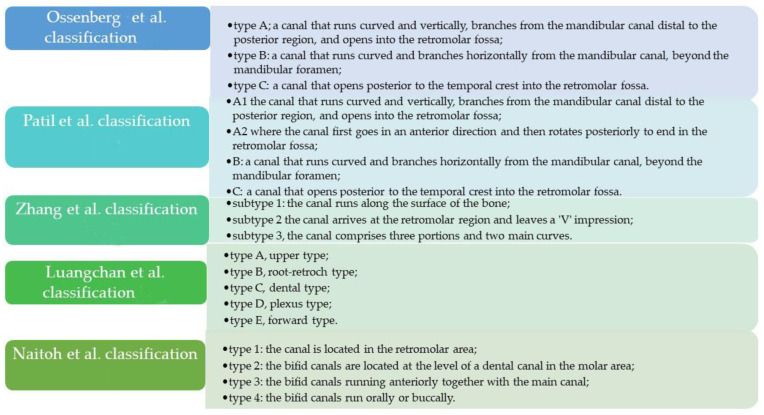
Diagram of the different classifications [12,13,15,16,17].

The clinical implications of the presence of a bifid canal include failure of anesthesia in the posterior area of the mandible, neurovascular damage of the retromolar canal, bleeding, changes in the sensitivity of the involved area, and a pathway for the spread of infectious or tumor processes [9,10,11]. These clinical implications have been valid reasons why authors have attempted to describe the presence of these canals over the years, using different classifications (Figure 1) [12,13,14].

Ossenberg [12] and von Arx et al. [14] proposed to classify bifid and trifid canals into three categories: type A includes a canal that runs curved and vertically, branches from the mandibular canal distal to the posterior region, and opens into the retromolar fossa; type B includes a canal that runs curved and branches horizontally from the mandibular canal, beyond the mandibular foramen; and type C includes a canal that opens posterior to the temporal crest into the retromolar fossa. Patil et al. further divided one of these anomalies into two variants [13]. Specifically, they grouped type A of Ossenberg’s classification into two variants: A1, where the canal goes posteriorly and superiorly and opens into the retromolar fossa, and A2, where the canal first goes in an anterior direction and then rotates posteriorly, to end in the retromolar fossa.

Zhang et al. proposed three subgroups: subtype 1, which runs along the surface of the bone; subtype 2, which arrives at the retromolar region and leaves a V impression; and subtype 3, which comprises three portions and two main curves. It simulates a U when reaching the retromolar region [15].

Luangchan et al. classified the variation into five categories: type A, upper type; type B, root-retroch type; type C, dental type; type D, plexus type; and type E, forward type [16].

**Figure 2:**
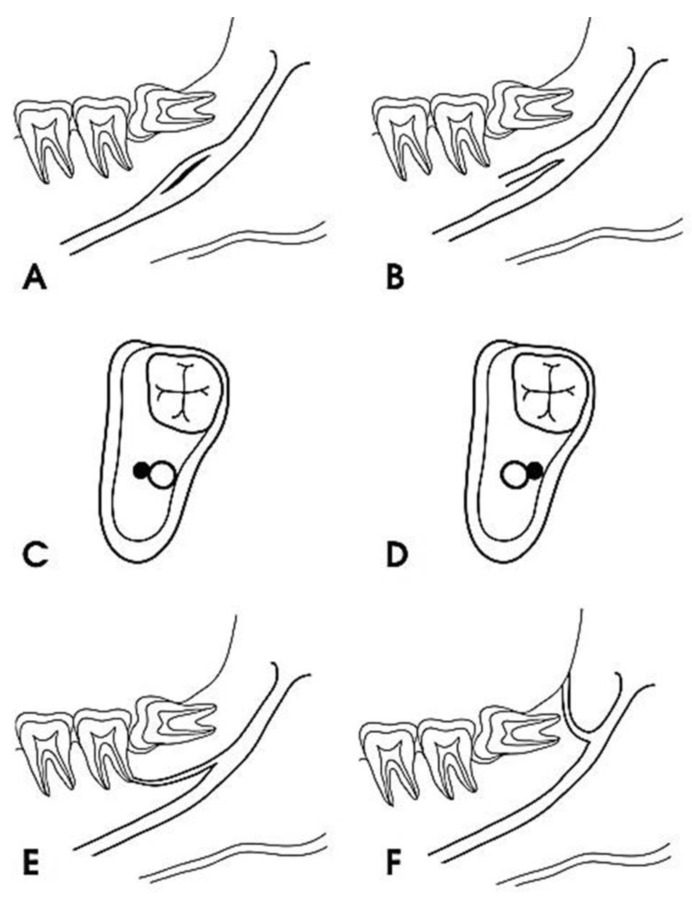
Naitoh classification. The forward canal divided into one with confluence (**A**) or one without confluence (**B**). The bucco-lingual canal from the buccal or lingual wall (**C**,**D**). The dental canal reaches the root apex (**E**). The retromolar canal opens into the retromolar region (**F**). Reprinted with permission from [18] Copyright (2019).

According to the Naitoh et al. [17] classification, the bifid canal can be classified into type 1 when it is located in the retromolar area, close to the cortical bone; type 2 when the bifid canals are located at the level of a dental canal in the molar area (and for this, we distinguish three subclasses depending on whether the first, second, or third molar is involved); type 3 when the bifid canals run anteriorly together with the main canal and have subclasses depending on whether the bifid canals join the main canal during their course; and type 4 bifid canals, running orally or buccally (Figure 2).

Clinical implications highlight the importance of assessing the bifid mandibular canal through radiological imaging, including orthopantomogram (OPG) and cone-beam computed tomography (CBCT). Zhang et al. (2018), when evaluating the panoramic radiographs of patients with bifid mandibular canals detected in CBCT, observed that only 7.1% of the bifid mandibular canals were visible in the panoramic views and, therefore, concluded that this imaging method was unreliable [15]. Shah et al. (2018) also found that the sensitivity of panoramic radiography in terms of identifying bifid mandibular canals was only 11% [19].

For a correct and reliable pre-surgical assessment of the inferior alveolar canal, it is necessary to perform CBCT rather than panoramic radiography to appreciate each bone variation [20].

Due to the previous anatomical and clinical considerations, this study aimed to assess the prevalence and configuration of bifid mandibular canals within a European adult population using CBCT.

## 2. Materials and Methods

The population study included patients attending the Clinical Dental Services of the Department of Innovative Technologies in Medicine & Dentistry of the G. d’Annunzio University of Chieti-Pescara. The sample included 300 subjects (600 hemi-mandibulae). The analyses were conducted on CBCTs from the clinic’s archives and performed between 2012 and 2021. The study was approved on 16 December 2021, as No. 27, by the Inter-Institutional Ethics Committee of the G. d’Annunzio University of Chieti-Pescara. The CBCTs of the patients who gave their consent to the use of the radiograms for the study were included.

CBCT scans were obtained using PaX-Zenith3D (Vatech, 13, Samsung 1-ro 2-Gil, Hwaseong-si, Gyeonggi-do, 445-170, Korea). Although recent studies indicate that small variations in the head position do not affect the accuracy of 3D CBCT measurements [19], each scan obtained is standardized and performed by trained personnel. Patients were seated, stabilized with head straps and chin rests, and monitored to ensure that they remained motionless for the duration of the scan (approximately 50 s).

Using the Ez3D plus Premium software ver. 1.2.6.2 (Vatech, 13, Samsung 1-ro 2-Gil, Hwaseong-si, Gyeonggi-do, 445-170, Korea), axial, sagittal, transverse, and panoramic images were reconstructed for all jaws, and 3D reconstructions were used when necessary.

The observations and measurements in the present study followed the protocol described in the study by Orhan et al. [21]. Briefly, the paths and lengths of bifid mandibular canals were measured in sagittal sections or reconstructed panoramic images using the specific software function that allows the observer to measure both linear and curved structures.

**Figure 3:**
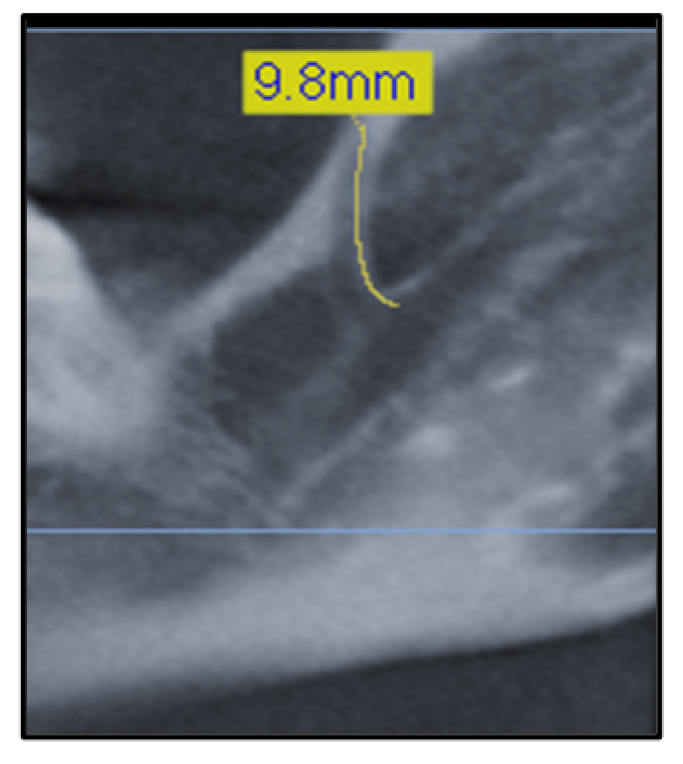
Measuring the length of a Type 1 canal.

The length of the mandibular canal was measured from the point of separation from the main canal to the terminal point in the mandible (Figure 3).

**Figure 4:**
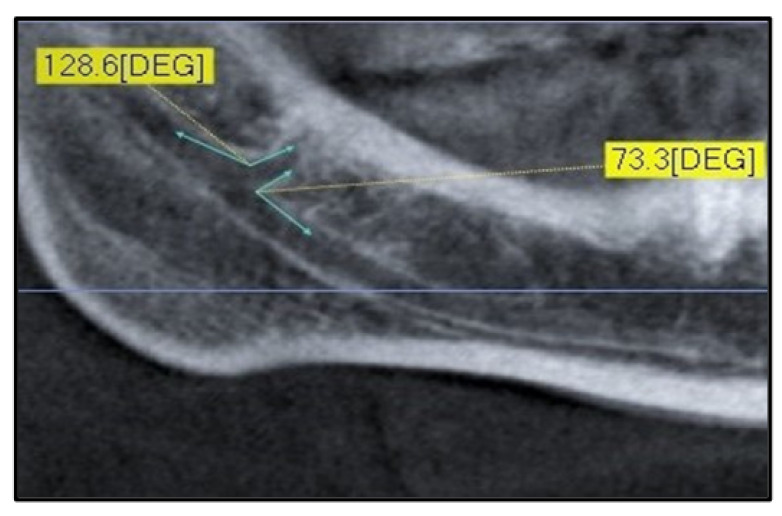
Angle measurement of a Type 1 canal.

The upper and lower angles between the canals were also measured using CBCT with software processing of sagittal or panoramic sections (Figure 4).

The upper angle is defined as the angle formed between the main canal and the upper wall of the mandibular bifid canal. In contrast, the lower angle is defined as the angle between the main canal and the lower wall of the mandibular bifid canal.

**Figure 5:**
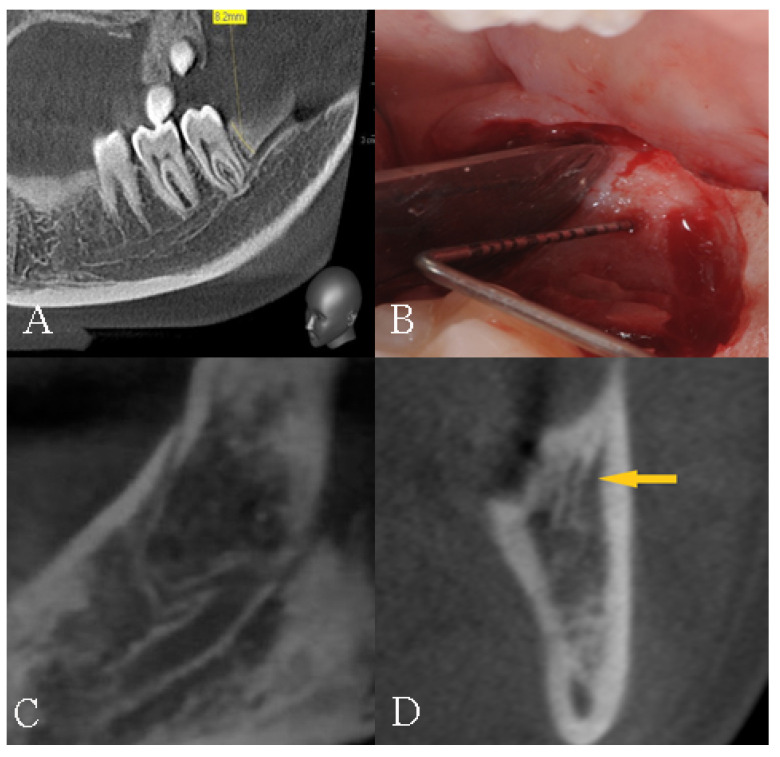
Type 1, retromolar canal: (**A**) sagittal view; (**B**) intraoperative view; (**C**) Panorex reconstruction; and (**D**) cross-section reconstruction. Arrow indicated the presence of the canal.

**Figure 6:**
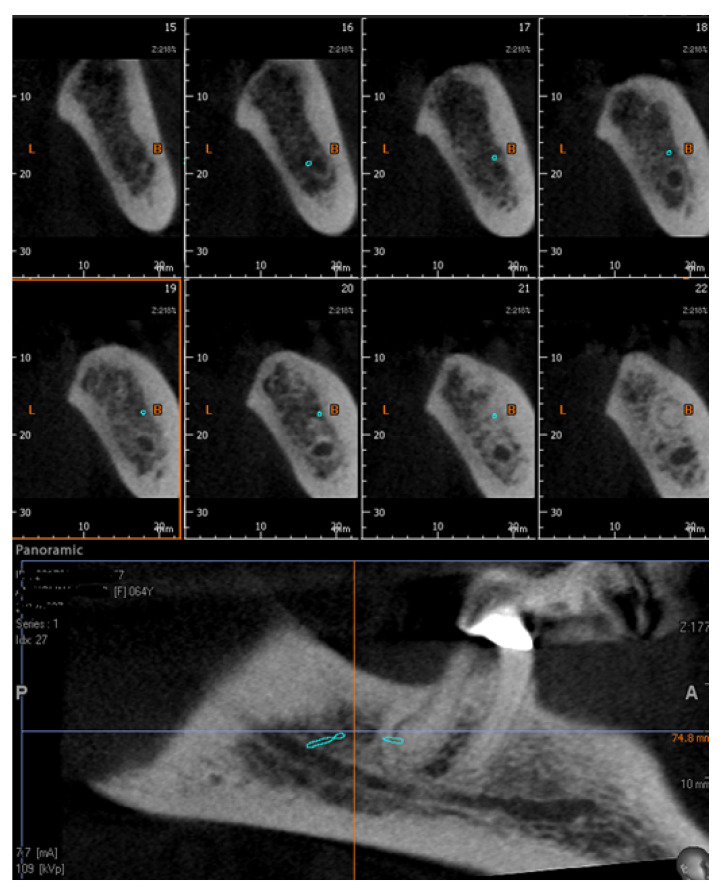
Type 2, dental canal: cross and Panorex reconstructions. Reconstruction shows arrival at the apex of the 4.7 tooth element.

**Figure 7:**
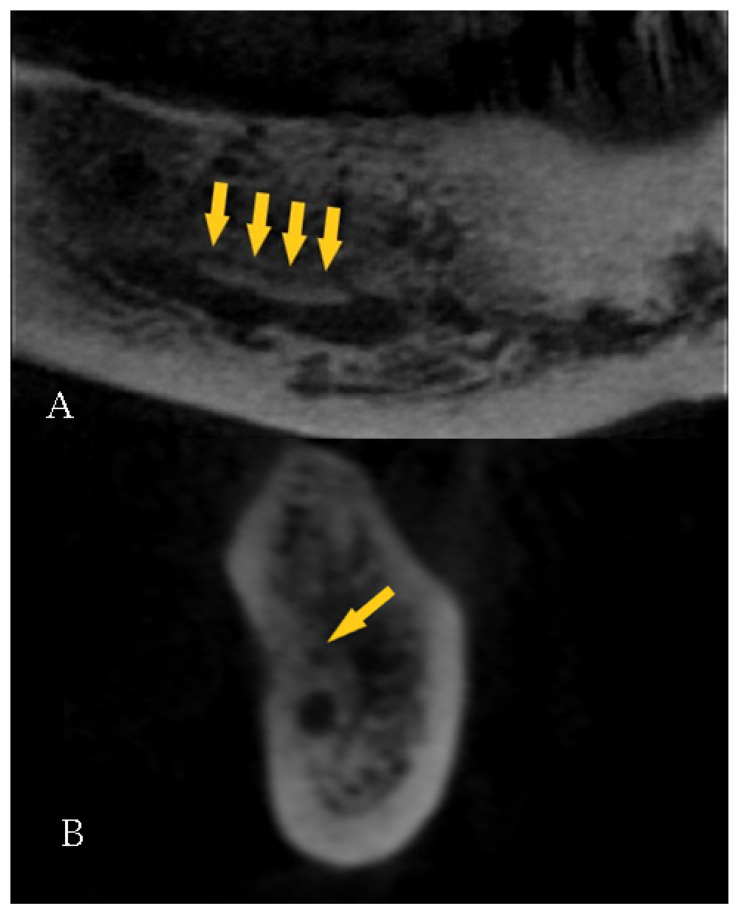
Type 3, confluent anterior canal: (**A**) Panorex and (**B**) cross-section reconstructions. Arrows indicate the presence of the canal.

**Figure 8:**
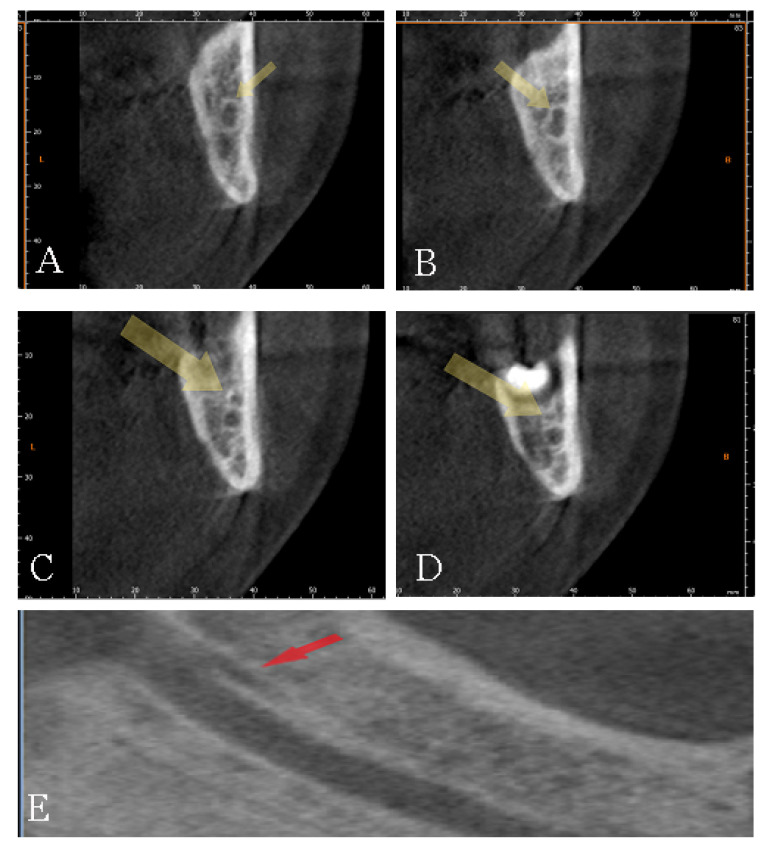
Type 3, not confluent canal: (**A**–**D**) cross-section reconstructions and (**E**) Panorex reconstructions. Arrows indicate the presence of the canal.

**Figure 9:**
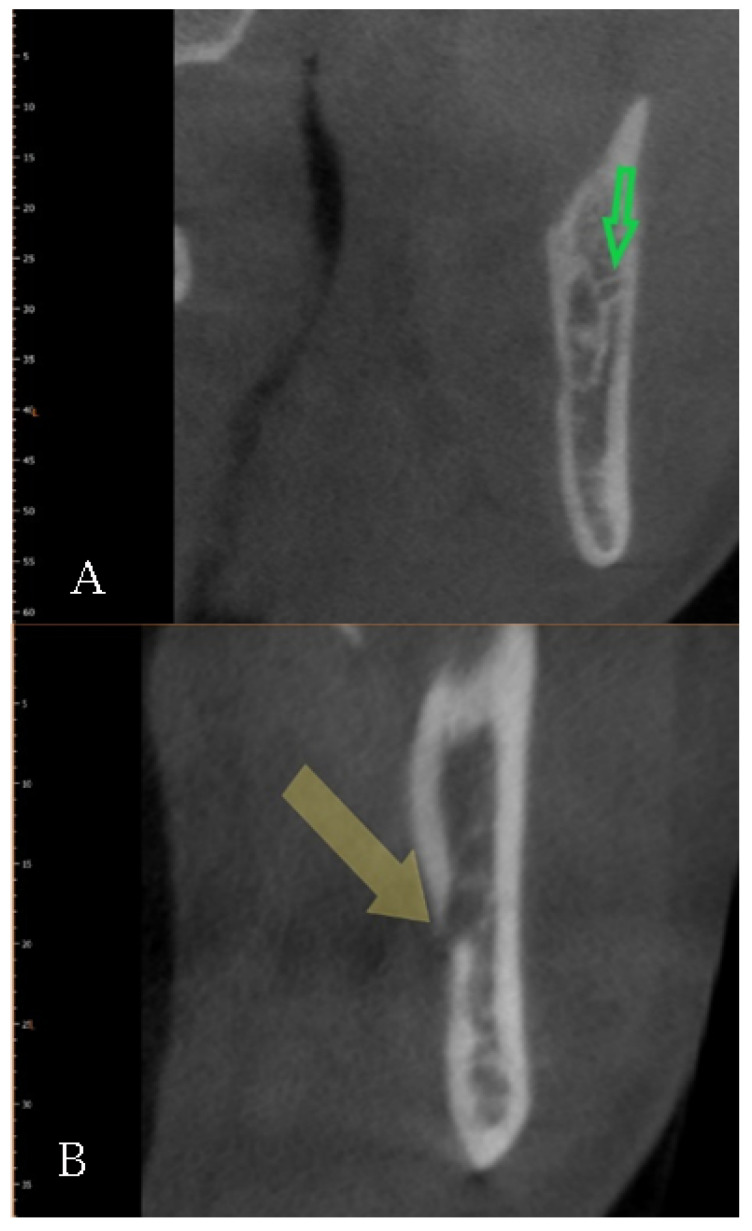
Type 4: (**A**) buccal canal cross-section reconstructions and (**B**) lingual cross-section reconstructions. Arrows indicate the presence of the canal.

The data were collected by a well-experienced radiologist (B.F) and a well-experienced dental clinician (G.V.). In the case of a disagreement, a well-experienced anatomist (S.B.) was consulted for clarification. The mandibular bifid canals were classified according to their position and placed within four main groups according to the Naitoh et al. classification [22], namely: retromolar (Figure 5), dental (Figure 6), anterior (Figure 7 and Figure 8), and buccolingual (Figure 9).

Anterior canals were further divided into confluent and non-confluent canals; dental canals were further divided into first, second, and third molar canals; oro-vestibular canals were divided into vestibular and oral canals (Table 1).

Statistical analyses were performed using SPSS software (SPSS vers. 26, Chicago, IL, USA). The tests performed were Pearson’s chi-square to assess the categorical variables (sex and location) and Student’s *t* test to assess the parametric variables (age, lengths, and angles).

The *p*-value was considered significant for a value less than or equal to 0.05.

## 3. Results

The sample size included 300 subjects (600 hemi-mandibles), of whom 147 (49%) were males and 153 (51%) were females. The mean age of the patients was 47.07 ± 17.7 years (range 18–89). Anatomical variants of the mandibular canal were identified in 173 out of 600 sides (28.8%) and 151 out of 300 patients (50.3%). In 22 subjects, anatomical variants were present bilaterally (7.3%). Regarding gender, they were observed in 74 out of 153 female patients (48.4%) and in 77 out of 147 male patients (52.4%).

As shown in Table 2, the most frequently encountered type of bifid canal was Type 3 (*n* = 70 (40.5%), 43 on the right side (61.4%) and 27 on the left side (38.6%)), followed by the Type 1 canal (*n* = 68 (39.3%), 31 on the right (45.6%) and 37 on the left (54.4%)), Type 2 canal (*n* = 25 (14.5%), 14 identified on the right side (56%) and 11 on the left side (44%)), and the Type 4 canal (*n* = 10 (5.9%), 4 on the right (40%) and 6 on the left (60%)). Of the 70 anterior canals identified (Type 3), 36 (51.5%) were with confluence and 34 (48.5%) without confluence. Of the 25 dental canals identified, 3 (12%) extended to the root apex of the first molar, 10 (40%) to the second molar, and 12 (48%) to the third molar. Of the 10 buccal canals, 5 (50%) were identified as buccal and 5 (50%) as lingual. All Type 1 canals ended in the retromandibular region distal to the teeth. The bifid canals’ average length on the mandible’s right side was 11.96 ± 5.57 mm, compared to 11.38 ± 4.89 mm for those measured on the left side.

Considering the measurements per canal type, the average length of the retromolar canals (type 1) was 11.7 ± 3.95 mm (right side 11.38 ± 4.11 mm; left side 12.02 ± 3.78 mm). The average length of the dental canals (Type 2) was 8.69 ± 3.71 mm, with the right side measuring 8.41 ± 3.06 mm and the left side measuring 8.97 ± 4.37 mm. The anterior canals (Type 3) had an average length of 13.75 ± 5.51 mm (right side 14.36 ± 5.86 mm; left side 13.15 ± 5.17 mm), and the buccal canals (Type 4) had an average length of 3.47 ± 0.82 mm (right side 3.12 ± 0.94 mm; left side 3.83 ± 0.70 mm).

As shown in Table 3, the upper mean angles measured 123.71 ± 24.30 degrees on the right side and 118.1 ± 29.1 degrees on the left side, while the lower mean angles were 56.03 ± 25.56 degrees on the right side and 62.75 ± 32.46 degrees on the left side. The right upper mean angles of the retromolar (Type 1) measured 116.97 ± 22.11 degrees, the dental (Type 2) measured 108.25 ± 15.52 degrees, the anterior (Type 3) measured 136.35 ± 22.25 degrees, and the oro-vestibular (Type 4) measured 94.17 ± 4.05 degrees. As regards the lower angles, Type 1 measured 61.48 ± 21.89 degrees, Type 2 measured 65.04 ± 12.35 degrees, Type 3 measured 43.92 ± 17.58 degrees, and Type 4 measured 112.37 ± 5.89 degrees. The left upper mean angles of the retromolar canal measured 112.89 ± 29.59 degrees, those of the dental canal measured 104.4 ± 25.05 degrees, those of the anterior canal measured 136.18 ± 23.09 degrees, and those of the oro-vestibular canal measured 93.35 ± 2.72 degrees. The mean lower angles of the retromolar canal measured 66.28 ± 33.64 degrees, those of the dental canal 69.98 ± 24.26 degrees, and those of the anterior canal measured 45.59 ± 25 degrees.

Student’s *t* tests on the lengths of the two sides, angles, and age resulted in a *p*-value > 0.05 (Table 4). In Pearson’s chi-square test, however, the possible relationships between gender and location did not find any statistical association.

## 4. Discussion

### 4.1. CBCT as a Reliable Preoperatory Assessment Method

The presence of accessory foramina in the posterior region of the mandible highlights the clinical relevance of the neurovascular supply in this area. Although the only structure commonly considered is the mandibular canal, easily detected by modern CT scans, the documented retromandibular canal and accessory foramina indicate a more complex neurovascular anatomy, which should be carefully investigated in the preoperative phases [23].

In this study, the evaluation of the incidence and the location of bifid mandibular canals in a European adult population showed the occurrence of this variation. Therefore, identifying anatomical variations of the mandibular canal is crucial in all surgical interventions that may involve it. The assessment of the retromolar canal and the dental canal is important, for example, during the bone harvesting procedure, when precisely the retromolar region is used as a donor site. In addition, the retromolar canal is close to the third molar, which exposes it to the risk of damage during surgery [10]. However, there is disagreement in the literature on the use of the CBCT in the preoperatory stages in cases of third molar extraction [24]. Several studies have highlighted the limitations of using the OPG to identify bifid mandibular canals, due to adjacent structures [25,26,27]. The studies where OPG was used as a diagnostic tool confirmed this limitation with results that the variation presence did not exceed 1%. Furthermore, according to some studies, the mandibular canal itself has been identified in only one in four OPGs [28,29,30]. The OPG can produce false-negative and false-positive results, as stated in the study by Elnadoury et al., due to the presence of airways and the appearance of the mylohyoid nerve imprint [31].

Modern volumetric imaging allows us to identify the presence of these variations. The use of CBCT has significantly improved the ability to assess accessory canals: as previously reported [21,22,29], the incidence rates of anatomical variations in the mandibular canal exceed 60%. In addition, the high incidence of bifid canals indicates a higher risk of clinical and surgical complications due to a neurovascular lesion affecting these structures [21,29].

### 4.2. Bifid Canals: A Difficult Classification, Established Presence, and Clinical Implications of the Morphometric Data

Our study used the classification proposed by Naitoh et al. [17], which divides the canals into four groups, developed and reported in their study using CBCT radiograms.

However, a more recent review on the gross anatomy of the bifid/trifid canals clarifies the definition of retromolar canal as a regular branch of the mandibular canal and recognizes as the bifid canal “an intramandibular canal running parallel to the mandibular canal with/without confluence to the main canal” [32]. This new revision and classification, published after our study performance, assumes that those canals defined as subtypes of bifid canals host additional branches of dental neurovascular bundle.

In our study design, the Naitoh et al. [17] classification appeared the most complete and therefore was used to classify the data. In our study, the presence of bifid mandibular canals was 50.3%, slightly in disagreement with recent data in the literature: Naitoh et al. [17] reported a prevalence of 65%; Orhan et al. [21] and Zhou et al. [33] reported a prevalence of 66.5% and 42.53%, respectively; and Elnadoury et al. [31] reported a prevalence of 34%.

The trend is confirmed when considering the total number of hemimandibles: in our study, the percentage frequency was 28.8%, while the prevalence in Naitoh et al.’s [17] study was 43%; in Orhan et al.’s [21] study, it was 46.5%; and it was 16% in Zhou et al.’s study [33]. Both Zhou et al. [33] and Elnadoury et al. [31] speculated on such a variety of prevalence in the literature by focusing on two key factors: the radiographic examination methods and the different ethnic populations examined. De Olivera et al. [34] reported a prevalence of 19% in Belgium, Muinelo-Lorenzo et al. [20] reported a prevalence of 36.8% in Spain. Furthermore, Kang et al. [35] reported a prevalence ranging from 10% to 22.6% in South Korea, and Fu et al. [36] reported a prevalence of 30.6% in Taiwan.

In our study, there were statistically significant differences between male and female subjects, in line with the data reported by Orhan et al. [21] as well as Elnadoury et al. [30].

Regarding canal type data, the prevalence rates of retromolar bifid channels (Type 1) in our study were 22.3%, in line with the studies of Naitoh et al. [17] (25.4%) and Orhan et al. [21] (28.1%) but in disagreement with the studies of Zhou et al. [33] (40%) and Elnadoury et al. [31] (7.5%).

As far as Type 2 canals are concerned, the percentage in our study coincided with that found by Orhan et al. [21], i.e., 8.3%, and differed little from the results of Naitoh et al. [17] (7.4%) and the results of Zhou et al. [33] (10.48%) and Elnadoury et al. [31] (6.4%).

Concerning Type 3 canals, the data were in line with those by Orhan et al. [21] (26.9%) but different from the data of the studies by Naitoh et al. [17] (44.3%), Zhou et al. [33] (46.67%), and Elnadoury et al. [31] (14.6%). Finally, the percentage of buccolingual canal frequency (3.4%) differed from the data in the literature; the percentage found in our study was 3.4%, while in Naitoh et al. [17], the percentage was 1.6%; in Orhan et al. [21], it was 9.9%; it was 2.8% in Zhou et al. [33]; and it was 1.4% in Elnadoury et al. [31].

Our study evaluated both the average lengths of right and left bifid mandibular canals and the average length per canal type. The average lengths for all canal types ranged from 17.53 mm to 6.39 mm on the right and from 16.27 to 6.49 mm on the left, without any statistically significant difference between right and left sides. However, different lengths were found for each type of canal. In particular, the longest mean length was of the anterior canal (Type 3) on both the right and the left (20.22 mm and 18.32 mm, respectively), while the shortest length was of the vestibular canal (Type 4) on both the right and the left (2.18 mm and 3.13 mm, respectively). Our results agree with those of the study by von Arx et al. [37], and this agreement could be attributed to the measurement method. However, Puche-Roses et al. pointed out that these data are derived from a bidimensional measurement that does not assess the three-dimensional intraosseous canal path, unlike the same authors who used helical CT scans [38]. Our study also assessed the lower-right and/or upper-right and lower-left and/or upper-left bifurcation angles of bifid mandibular canals from the main canal. Notably, no significant differences were found between the angle measurements of the various canal types. Our result seems to agree with the data reported by the different research groups in the von Arx study [37]. In Rahsuren et al.’s study, no statistically significant differences were found between the upper angles of each type [39]. However, about the lower angles, a statistically significant difference was found between the retromolar and dental canals and between retromolar and trifid canals. In the studies by Zhang et al. [15] and Orhan et el. [21], there were no statistically significant differences between the right- and left-side angles. It is important to emphasize that the closer the angles are to 90°, the sharper they are, and from a clinical point of view, there is an increased likelihood of contact with the canal itself during clinical maneuvers.

The limits of the study are represented by the sample sizes and by the measures relying on the experience and the abilities of the operators.

The established presence of bifid mandibular canals and their neurovascular content provide fundamental explanations for complications during clinical practice in those interventions involving the mandible, such as implant placement, extraction of the included third molar, osteotomy of the divided sagittal branch, and failure in anesthesia. The failure of inferior nerve anesthesia, with the permanence of dental pain during extraction or surgical removal of impacted mandibular third molars, is called the “escape pain phenomenon”; this pain is due to the nerve content of the retromolar canal, which innervates the retromolar triangle mucosa, buccal mucosa, and buccal gingiva of the mandibular premolar and molar regions [11] and requires a different anesthetic technique, such as the Gow-Gates technique [33]. Other surgical complications due to failure to accurately localize a bifid canal include intra- and postoperative bleeding, the altered sensation of the involved area [40], traumatic neuroma, and hematoma formation [15,19].

There are case reports in the literature documenting severe and persistent pain after implant placement in the posterior mandible, despite the adequate anatomical host sites in the panoramic radiograph [41,42]. After CBCT imaging, it was confirmed that the implants interfered with bifid canals and a significant reduction in pain was noted following the removal of the implants. A severe hemorrhagic complication related to the presence of a bifid canal was reported by Verea linares et al. [43]. A partially erupted third molar, closely associated with a bifid canal, was treated with coronectomy. Following the failure of measures to locally stop the bleeding, the patient required bleeding control under general anesthesia. The authors suggested performing a CBCT for appropriate surgical planning in a suspected bifid canal. Furthermore, they recommended complete removal of the tooth rather than coronectomy to allow direct access to the source of bleeding [43].

Other clinically relevant complications are related to endodontic treatments and include over-instrumentation and/or extrusion of filling materials. The latter can directly or indirectly damage the mandibular canals’ neurovascular structures via a bifid mandibular canal. This will lead to severe complications, including excruciating pain, changes, or loss of sensation, and/or necrosis of the skin or the mucosa (Nicolau syndrome) [44,45,46].

Finally, further clinical implications of the presence of bifid mandibular canals may be localized pain in the retromolar region in the case of patients with partial prostheses pressing on the bundle from the retromolar foramen [25] or their possibility of acting as a pathway for the spread of cancer or infectious processes [33].

## 5. Conclusions

In conclusion, the results of our study indicate that a bifid mandibular canal is a relatively common anatomical variation of the mandibular canal and preoperative CBCT analysis is recommended for accurate evaluation to avoid and reduce intraoperative and postoperative complications. Furthermore, its assessment is fundamental to better plan not only endodontic and surgical but also anesthetic treatments directed at the posterior portion of the mandible. Any unexplained occurrence of altered sensitivity or formation of hemorrhages and/or hematomas may be caused by the presence of a radiographically visible bifid mandibular canal that contains neurovascular structures.

## Figures and Tables

**Table 1:** Classification according to Naitoh et al. [17].

Type 1	Type 2	Type 3	Type 4
Retromolar	Dental	Forward	Buccolingual
	First molar (A)	With confluence	Buccal canal
	Second molar (B)	Without confluence	Lingual canal
	Third molar (C)		

**Table 2:** Prevalence of bifid mandibular canals based on gender, type, and location.

Classification	Tot. Pat.	Tot. Sides	Right Side			Left Side		
	300 (%)	600 (%)	M. *n* (%)	F. *n* (%)	Tot *n* (%)	M. *n* (%)	F. *n* (%)	Tot *n* (%)
**Type 1** Retromolar	22.7	11.3	19 (6.3)	12 (4)	31 (10.3)	18 (6)	19 (6.3)	37 (12.3)
**Type 2** Dental	8.3	4.2	8 (2.7)	6 (2)	14 (4.67)	8 (2.7)	3 (1)	11 (3.67)
First molar	1	0.5	0	2 (0.7)	2 (0.7)	1 (0.3)	0	1 (0.3)
Second molar	3.3	1.7	3 (1)	1 (0.3)	4 (1.3)	4 (1.3)	2 (0.7)	6 (2)
Third molar	4	2	5 (1.7)	3 (1)	8 (2.7)	3 (1)	1 (0.3)	4 (1.3)
**Type 3** Front	23.3	11.7	27 (9)	16 (5.3)	43 (14.3)	11 (3.7)	16 (5.3)	27 (9)
Confluent	12	6	16 (5.3)	9 (3)	25 (8.3)	3 (1)	8 (2.7)	11 (3.7)
Not confluent	11.3	5.7	11 (3.7)	7 (2.3)	18 (6)	8 (2.7)	8 (2.67)	16 (5.3)
**Type 4** Buccolingual	3.3	1.7	1 (0.3)	3 (1)	4 (1.3)	1 (0.3)	5 (1.7)	6 (2)
Buccal	1.7	0.8	0	2 (0.7)	2 (0.7)	0	3 (1)	3 (1)
Lingual	1.7	0.8	1 (0.3)	1 (0.3)	2 (0.7)	1 (0.3)	2 (0.7)	3 (1)

List of abbreviations: Tot. = total; Pat. = patients; M. = male; F = female.

**Table 3:** Age and length of left and right sides.

	N Cases	Minimum	Maximum	Average	Standard Deviation
**Age (years)**	300	18	89	47.07	17.71
**Length right (mm)**	92	1.80	28.30	11.96	5.56
**Ang. sup. right (°)**	92	22.50	162.20	123.70	24.30
**Ang. inf. right (°)**	92	11.90	130.40	56.02	23.55
**Length left (mm)**	81	2.70	19.60	11.37	4.88
**Ang. sup. left (°)**	81	35.60	167.00	118.05	29.00
**Ang. inf. left (°)**	81	8.70	154.30	62.75	32.45

List of abbreviations: Ang. = angle; sup. = superior; inf. = inferior.

**Table 4:** Student’s *t* test.

	Average	No.	Standard Deviation	Average Standard Error	Sign. (*p* Value)
Right length	11.38	22	3.62	0.77	0.371
Long left	11.79	22	5.26	1.12	
Top-right corner	117.97	22	26.76	5.70	0.790
Top-left corner	125.99	22	22.27	4.74	
Ang. inf. right	57.57	22	18.02	3.84	0.628
Ang. inf. left	50.50	22	16.09	3.43	
Age	47.01	92	19.33	2.01	0.765
Right length	11.96	92	5.56	0.58	
Age	48.33	81	16.76	1.86	0.904
Long left	11.37	81	4.88	0.54	
Age	47.01	92	19.33	2.01	0.160
Top-right corner	123.70	92	24.30	2.53	
Age	47.01	92	19.33	2.01	0.076
Ang. inf. right	56.02	92	23.55	2.45	
Age	48.33	81	16.76	1.86	0.311
Top-left corner	118.05	81	29.00	3.22	
Age	48.33	81	16.76	1.86	0.298
Ang. inf. left	62.75	81	32.45	3.60	

## Data Availability

Data are available upon reasonable request to the corresponding author.
